# Associated uncertainty estimation during the validation process of TCID_50_ and FRNT neutralization assays against SARS-CoV-2 variants, used in population surveillance research and correlates of protection

**DOI:** 10.3389/fimmu.2026.1768395

**Published:** 2026-03-18

**Authors:** Edgar Reyna-Rosas, Milton Nieto-Ponce, Andrea Palencia-Reyes, Carlos Blancas-Ruíz, Claudia Carranza, Martha Torres, Horacio Zamudio-Meza

**Affiliations:** Laboratorio de Inmunobiología de la Tuberculosis, Instituto Nacional de Enfermedades Respiratorias Ismael Cosío Villegas, Mexico City, Mexico

**Keywords:** correlates of protection against SARS-CoV-2, FRNT, method validation, TCID50MN, uncertainty estimation

## Abstract

Neutralizing antibody (nAb) assays are essential for evaluating antibody-mediated immune response against SARS-CoV-2, in the context of efficacy and potency of vaccines, as well as population-level surveillance. This study aimed to estimate the associated uncertainty during the validation of two neutralization assays—50% tissue culture infectious dose microneutralization (TCID_50_MN) and fluorescent focus reduction neutralization test (FRNT)—to measure activity of nAbs against SARS-CoV-2 variants of concern (VOCs) within an ISO/IEC 17025:2017 accredited laboratory. Human serum samples were tested against five SARS-CoV-2 variants (Wuhan, BA.2, BA.5, XBB.1, and JN.1) using vesicular stomatitis virus (VSV) pseudotyped viruses. The validation process was based on international guidelines for the suitability and validation of analytical methods. Both assays demonstrated high diagnostic performance to detect neutralizing activity, where FRNT yielded higher sensitivity but greater inter-analyst variability than TCID_50_MN. These results highlight the importance of identifying the level of impact of factors associated with obtaining a result, such as the training of analysts and the standardization of the test. This study establishes a validated framework for nAb quantification in compliance with international standards. The declaration of associated uncertainty to each method, along with sustained internal quality control, strengthens the diagnostic capacity of these assays in clinical research of correlates of protection and immunity induced by vaccination, in addition to reinforcing transparency and regulatory compliance, as critical elements in decision-making.

## 1 Introduction

Three months after the first case was reported in Wuhan, China, the World Health Organization (WHO) declared coronavirus disease 2019 (COVID-19) as a pandemic in 2020. It is an infectious disease caused by severe acute respiratory syndrome coronavirus type 2 (SARS-CoV-2); it can present in five clinical forms: (1) asymptomatic infection; (2) mild disease; (3) moderate disease; (4) severe disease; and (5) critical disease (1). This virus is a betacoronavirus with a positive-sense single-stranded RNA genome, composed of four structural proteins: envelope protein (E), membrane protein (M), nucleocapsid protein (N), and spike protein (S). The latter is responsible for binding to the angiotensin-converting enzyme 2 (ACE2) receptor, thereby initiating the replication cycle, and is the main therapeutic target (1, 2).

As part of the strategies to contain and mitigate the catastrophic effects caused by the disease worldwide, prior knowledge about the coronavirus (SARS-CoV), as well as the technologies implemented in other vaccines, allowed the development of vaccines against SARS-CoV-2 in record time. Currently, 10 vaccines have been approved in Mexico by the Federal Commission for Protection against Sanitary Risks (COFEPRIS) for emergency use, 2 of which has complete sanitary registration (3, 4). Simultaneously, it is necessary to have well-established assays to evaluate effectiveness in terms of the protective immune response of vaccines (5, 6). Therefore, evaluating neutralizing antibodies (nAbs) is a key component, as these have the capacity to bind to the surface of viral particles, reducing their spread to other cells and tissues, in addition to immunological memory, which provides protection against subsequent infections, and can be used as an element in decision-making regarding the effectiveness of vaccines (7, 8).

The evaluation of nAb activity in serum samples is performed using different methodologies such as the plaque reduction neutralization test (PRNT) or the microneutralization (MN) assay with viral isolates, with the former being the gold standard (9). Both involve making serial dilutions of the sample and a known concentration of virus in cells permissive to infection. At the end of the assay, a visual analysis of the cells is performed to determine the degree of protection (10). However, when employing isolates of active pathogens such as SARS-CoV-2, greater infrastructure is required, such as specialized laboratories with enhanced biosecurity measures, hence the need to generate biotechnological alternatives to solve this technical problem (11, 12).

The construction and production of recombinant pseudotyped viruses that express the SARS-CoV-2 spike protein on their surface emerges as a safe alternative that allows nAb evaluation in terms of biosafety, obtaining results comparable to those obtained with the complete virus without losing sensitivity and specificity. Additionally, it offers a versatile analytic platform, such as employing reporter genes like enhanced green fluorescent protein(eGFP) or luciferase (13), which implies a technological challenge, since this requires the use of specialized laboratory equipment. On the other hand, the conventional MN assay only requires microscopical analysis to evaluate the neutralizing capacity of serum samples in cell monolayers, in which the presence or absence of cytopathic effect is registered (14).

The international regulatory requirements, such as those established by the European Medicines Agency (EMA), requests the evaluation of immunogenicity of new vaccines, where the nAbs test is mandatory to determine the vaccine’s potency in terms of protective capacity. Additionally, it recommends that all experiments related to the clinical study be carried out in the same laboratory, since the intervention of two or more laboratories can increase the variability of study results (15). Furthermore, to ensure the reliability of the results issued, it is required to have international accreditations applicable to such laboratories, such as ISO/IEC 17025:2017, whose focus is to evaluate technical competence, alongside other requirements, among which experimental validation of assays and, importantly, the estimation of associated uncertainty.

The objective of assay validation is to demonstrate with objective and conclusive evidence that it fulfills the purpose for which it was designed (16–19). Such evidence is also necessary to comply with the Common Technical Document (CTD), which is a necessary requirement to register a pharmaceutical product for human use (20). On the other hand, associated uncertainty is a commonly used term in clinical laboratories or biochemical processes; it is a parameter associated with the result of a measurement that provides a dispersion interval around the true measured value. To estimate it, it is necessary to consider all possible systematic and random error sources involved in obtaining the result and finally multiply it by a coverage factor (*k*), which provides the interval (expanded uncertainty). While not a mandatory requirement and is rarely reported for this type of biological assay, estimating this parameter during method validation is fundamental to ensuring the reliability of the results that will be evaluated by regulatory bodies to approve or not the release of a drug and vaccine.

Before the health emergency caused by SARS-CoV-2, there were no laboratories accredited under the international standard ISO/IEC 17025:2017 in Mexico to develop an assay that evaluates nAbs activity with all the techno-scientific regulatory arguments. For this reason, in the present investigation, we present the results of the experimental validation process of two microneutralization assays using 50% tissue culture infectious dose microneutralization (TCID_50_MN) and fluorescent focus reduction neutralization test (FRNT) methods, up to uncertainty estimation, employed to evaluate the protective immune response in individuals vaccinated with a viral vector based on Newcastle disease virus (NDV) (21), being the first laboratory to obtain accreditation from the Mexican Accreditation Entity (EMA) starting in 2024 (accreditation number: Q-1844-287/24, https://catalogo.consultaema.mx:75/busqueda-laboratorios-de-ensayo) at the Latin American level, to evaluate the immune response induced by vaccination in the context of the TCID_50_MN assay.

## 2 Materials and methods

### 2.1 Cell line and pseudotyped viruses

For the development of this research, the Vero E6 cell line (CRL-1586. ATCC, USA) susceptible to SARS-CoV-2 infection was employed. These cells were maintained in Eagle’s Modified Essential Medium (EMEM, ATCC, USA) plus 10% fetal bovine serum (FBS, Gibco, USA) at 37 °C and 5% CO_2_ without antibiotics to reduce the long-term effect from their use. On the other hand, pseudotyped viruses based on replication-competent vesicular stomatitis virus (VSV), expressing on their surface the spike protein of the ancestral strain and Omicron subvariants BA.2, BA.5, XBB.1, and JN.1 of SARS-CoV-2, were provided by Dr. Sean P. J. Whelan from Washington University, USA (13). Each of them was propagated and titled in the same cellular system, generating several working banks, using previously validated methods in our laboratory.

### 2.2 Serum samples

Twenty human serum samples negative against SARS-CoV-2, collected before the COVID-19 pandemic, were used. Ten of them correspond to volunteers who presented a non-related respiratory viral infection and were collected during the H1N1 influenza epidemic in Mexico as part of research protocols approved by the Instituto Nacional de Enfermedades Respiratorias, Mexico, “Ismael Cosío Villegas”. Additionally, 20 serum samples obtained from individuals with a positive polymerase chain reaction (PCR) result for SARS-CoV-2 and three serum pools prepared from eight individuals with a positive PCR result for SARS-CoV-2 between 2022 and 2024 were used. In some samples, a previous analysis of total antibodies by the ELISA technique showed high nAbs titers for the ancestral Wuhan strain or the clinically relevant SARS-CoV-2 subvariants BA.2, BA.5, XBB.1 and JN.1, correlated with the data obtained by the microneutralization assay, from the ancestral Wuhan strain to the JN.1 subvariant. The latter has not completely disappeared and can be found in some regions of the world as part of the emerging variants under surveillance (24).

### 2.3 Personnel and equipment

The experimental validation of both assays was developed by two analysts on two different days, who demonstrated having documented technical competence under the international standard ISO/IEC 17025:2017, in addition to being part of the method research and development group. In both assays, single-channel micropipettes (Eppendorf, Germany) and multichannel micropipettes (Thermo Scientific, USA) were employed, in addition to a type A2 biosafety cabinet, refrigerators, ultra-freezers, and an incubator (Thermo Scientific), as well as an inverted optical microscope (VELAB, Mexico); in the case of the FRNT MN assay, the ImmunoSpot S6 ultimate M2 equipment (CTL, USA) was also used. All equipment had current maintenance, calibration, and qualification.

### 2.4 Validation parameters

Validation parameters were established according to the Validation of Analytical Procedures Q2 (R2) guidelines of the International Council for Harmonization of Technical Requirements for Pharmaceuticals for Human Use (ICH), the Eurachem guide, and the Guide to the Expression of Uncertainty in Measurement (16, 19, 22). The 40 serum samples with or without antibodies were employed to evaluate qualitative parameters such as selectivity, sensitivity, specificity, positive predictive value (PPV), negative predictive value (NPV), positive diagnostic confidence (PDC), and negative diagnostic confidence (NDC) using a 2 × 2 contingency table. Acceptance criteria for qualitative parameters were established as calculated chi-square test value > table value for the selectivity parameter; result ≥ 0.95 for sensitivity, specificity, PPV, NPV, and PDC parameters; result < 0.05 for the NDC parameter, while for repeatability and intermediate precision, the calculated geometric coefficient of variation (GCV) ≤ 30% was considered. Finally, parameters such as accuracy and robustness were evaluated with an acceptance criterion of recovery rate between 80% and 120%.

### 2.5 Pseudotyped virus titration

Pseudovirus titration was performed using two experimental approaches: the 50% tissue culture infectious dose (TCID_50_) technique, which considers damage to the cell monolayer, and fluorescent focus units (FFUs), evaluating the expression of the GFP reporter gene, both in a 96-well plate format, with Vero E6 cells at 1.3 × 10^4^ cells/well, seeded 24 ± 4 h prior to assay setup. To obtain TCID_50_, serial 1:10 dilutions of previously propagated viral stock were made up to 1 × 10^-7^ with EMEM medium, and 100 μL of each dilution was placed in wells with an equal volume of EMEM medium plus 2% FBS. Plate reading was performed 72 hpi with the help of the inverted optical microscope after fixing the cell monolayers with 3.7% formaldehyde solution (Sigma-Aldrich, USA) and washing with 1× PBS. TCID_50_/mL calculation was carried out using the Spearman–Karber formula; the results obtained were expressed in EC_50_ values and inhibitory dilution (23). On the other hand, to obtain the focus forming units (FFUs), an initial 1:10 dilution of viral stock was made, followed by seven serial 1:3 dilutions; 25 μL was placed on the plate with Vero E6 cells and incubated for 1 h ± 10 min at 37°C and 5% CO_2_. After the incubation time, 25 μL of semi-solid medium composed of EMEM medium + 1% FBS and 1% carboxymethylcellulose (CMC) (Sigma-Aldrich, USA) were added. After 24 ± 1 hpi, plates were fixed with 3.7% formaldehyde solution and washed with deionized water. Image capture and spot counting were carried out on the ImmunoSpot S6 ultimate M2 equipment with the following pre-established parameters: Filter changer control: AF488; lens control: position 5,000, range 2,000, precision 10, zoom 2.0990; image acquisition controls: no autoexposure, gain 13.467, time 1,150; smart count: diffuse processing normal, counted area 90%, manual gating 0.0010–9.6296 mm². FFU/mL values were calculated with the average of titers obtained per dilution.

### 2.6 TCID_50_ microneutralization assay

The technique reported by our research group was employed (21). As with titration, this assay used the 96-well plate format where 1.3 × 10^4^ cells/well of the Vero E6 line were seeded 24 ± 4 h before starting the assay. Serum samples were previously inactivated in a water bath at 56 ± 1 °C for 30 ± 5 min. Then, a first 1:10 dilution was made in EMEM medium, taking 20 μL to place them in the first row of the plate up to a final dilution of 1:131,220 with a dilution factor of 3. Pseudovirus stocks were used to prepare a viral suspension at a concentration of 2,500 TCID_50_/mL, and 40 μL was placed in all plate wells. The pseudovirus–serum mixtures were incubated for 1 h ± 10 min at 37 °C and 5% CO_2_ (neutralization process). Finally, this mixture was transferred to the plate with the cell culture monolayer, and reading was carried out 72 hpi. The neutralizing capacity of serum samples was expressed as the 50% effective concentration (EC_50_ in base 10 logarithm and inhibitory dilution) using the Spearman–Karber algorithm.

### 2.7 Fluorescent focus reduction neutralization test

In the same way as the TCID_50_MN assay, 96-well plates were seeded with 1.3 × 10^4^ cells/well of the Vero E6 line. Inactivated serum samples were diluted 1:10 with EMEM medium, up to a final dilution of 1:131,220 with dilution factor 3 for neutralization against the ancestral Wuhan strain and 1:5,120 with dilution factor 2 for neutralization against Omicron subvariants XBB.1 and JN.1. For dilution factor 2, pseudovirus suspensions were prepared at 8,000 FFU/mL, while for factor 3, a pseudovirus suspension was prepared at 4,000 FFU/mL. Twenty-five microliters of each solution was placed in each well to interact with each of the serum sample dilutions. The pseudovirus–serum mixtures were incubated for 1 h ± 10 min at 37 °C and 5% CO_2_. Then, 25 μL of the mixtures was transferred to the plate with cells, and incubation was repeated under the same conditions. After the time concluded, 25 μL of semi-solid medium was added, and image capture and counting were carried out 24 ± 1 hpi, fixing the plates with 3.7% formaldehyde solution and washed with deionized water. Image capture and spot counting were carried out as mentioned previously in pseudotyped virus titration section. Protection calculation was expressed as the 50% inhibitory concentration (IC_50_) calculated through a four-parameter non-linear regression (4PL).

### 2.8 Associated uncertainty estimation

We performed the calculation following the steps described in the Guide to the Expression of Uncertainty in Measurement. As a basis, we employed the mathematical models of the Spearman–Karber and 4PL test for the TCID_50_MN and FRNT methods, respectively. We estimated expanded uncertainty by variant and by analyst, using data from the intermediate precision assay. First, we determined which variables in each mathematical model were dependent and independent, then considered all possible sources of error that affect the reliability of the result, such as use of laboratory instruments and/or equipment, imperfections in corrections, or those associated human error. Then, we calculated the sensitivity coefficients for each mathematical model variable; that is, we obtained their partial derivatives, with which we obtained the combined uncertainty and finally multiplied by the coverage factor (*k*) to obtain the expanded uncertainty expressed in the same units of each method.

### 2.9 Statistical treatment

The results obtained from each test are expressed in EC_50_ for the TCID_50_MN assay, IC_50_ for the FRNT assay, and the protection dilution titer for both cases. The statistical treatment of the analytical performance parameters consisted of a contingency matrix and the chi-square test. For the rest of the analytical validation parameters, the statistical treatment consisted of obtaining the average, standard deviation (SD), and GCV%. With respect to intermediate precision, we applied a two-way analysis of variance (ANOVA) conducted with the help of the statistical program GraphPad Prism Ver. 10.1, according to the requirements of each aspect of the validation. For the calculation of the IC_50_ of the FRNT assay, we used the 4PL logistic regression test, designed to fit curves of dose–response experiments. Finally, we use the statistical data from analytical precision tests, the factors influencing the method obtained through a root cause analysis using the Ishikawa diagram, and the partial derivatives of each element of the mathematical model for the estimation of expanded uncertainty.

## 3 Results

### 3.1 Evaluation of analytical performance in the validation process

To determine the analytical performance of both assays and their ability to discriminate the inhibitory biological activity with both reading systems, we evaluated analytical parameters such as selectivity, sensitivity, specificity, PPV, NPV, PDR, and NDR for ancestral strain and subvariants considered in each assay. These analyses were done by one analyst on different days. In the TCID_50_MN assay, 20 serum samples without the presence of nAbs and 20 samples with the presence of nAbs against the ancestral strain of SARS-CoV-2 were evaluated, while for the FRNT assay, 10 serum samples negative against SARS-CoV-2 and 10 serum samples from positive PCR SARS-CoV-2 individuals previously analyzed with the presence of nAbs against the ancestral strain Wuhan and subvariants XBB.1 and JN.1 were evaluated. The samples were analyzed in duplicate, and the results obtained from the contingency matrix and the chi-square test showed 100% concordance; that is, negative samples did not present neutralizing activity, while positive samples did present neutralizing activity, meeting the established acceptance criterion, as it allows for clear discrimination between positive and negative samples in all cases with a high degree of reliability (**Table 1**).

**Table 1:** Summary of analytical performance parameters of TCID_50_MN and FRNT assays for the ancestral strain Wuhan.

Parameter	TCID_50_MN		FRNT	
	Result	Acceptance criteria	Result	Acceptance criteria
χ^2^	40	>3.842	20	>3.842
Sensitivity	1	≥0.95	1	≥0.95
Specificity	1	≥0.95	1	≥0.95
Positive predictive value (PPV)	1	≥0.95	1	≥0.95
Negative predictive value (NPV)	1	≥0.95	1	≥0.95
Positive diagnostic reliability (PDR)	1	≥0.95	1	≥0.95
Negative diagnostic reliability (NDR)	0	<0.05	0	<0.05

### 3.2 Repeatability and intermediate precision

The precision evaluation of both assays was expressed as repeatability and intermediate precision; three pools of serum prepared from eight individuals with positive PCR for SARS-CoV-2 between 2022 and 2024 that presented neutralizing activity against the ancestral strain and/or the Omicron subvariants BA.2, BA.5, XBB.1, and JN.1 of SARS-CoV-2 (previously calibrated with the international standard serum NIBSC: 20/136) were used. In the case of the TCID_50_MN assay, all five variants were evaluated, while the FRNT assay was performed using the ancestral strain Wuhan and Omicron subvariants XBB.1 and JN.1. Repeatability was evaluated by one analyst, while intermediate precision was assessed by two analysts on different days and expressed as GCV.

The three serum pools generated in our laboratory presented neutralizing activity against the ancestral strain at two quantification levels [high-level serum pool (HLSP) and low-level serum pool (LLSP)], allowing testing at three quantification levels with the ancestral strain: high, low, and medium; the medium level was obtained by diluting the HLSP by half [called medium-level serum pool (MLSP)] These were tested in both assays with 20 replicates. The remaining pool called V-LIT-Pool-3 was neutralizing also for Omicron subvariants BA.2, BA.5, XBB.1, and JN.1 at a single quantification level, so it was tested in both assays with 40 experimental replicates. Intermediate precision was assessed using the same experimental strategy as repeatability, but with a different analyst.

According to analytical validation guidelines, repeatability represents the distribution of results under specific conditions (16, 19). Repeatability results showed similar variability in both techniques, with a GCV difference between 0.42% and 4.28%. The assay with the smallest difference was performed with the Wuhan variant at the low quantification level, while the greatest difference was observed at the high quantification level. On the other hand, the assay with the BA.2 variant presented the highest GCV of all assays; however, this subvariant and BA.5 still need to be analyzed in the FRNT assay (**Table 2**).

**Table 2:** Repeatability results of TCID_50_MN and FRNT assays for the ancestral strain and the Omicron subvariants with different serum pools.

Spike SARS-CoV-2 ancestral strain Wuhan												
Sample	HLSP				MLSP				LLSP			
Type of assay	TCID_50_MN		FRNT		TCID_50_MN		FRNT		TCID_50_MN		FRNT	
Units	EC_50_	Dilution	IC_50_	Dilution	EC_50_	Dilution	IC_50_	Dilution	EC_50_	Dilution	IC_50_	Dilution
Geometric mean (MG)	3.18	1,497.96	3.37	2,332.12	2.88	755.96	2.99	974.20	2.45	283.14	2.54	345.18
GCV (%)	**18.17**		**13.89**		**14.43**		**16.30**		**14.69**		**15.11**	

Spike SARS-CoV-2, omicron subvariants												
	BA.2		BA.5		XBB.1				JN.1			
Sample	V-LIT-POOL-3											
Type of assay	TCID_50_MN		TCID_50_MN		TCID_50_MN		FRNT		TCID_50_MN		TCID_50_MN	
Units	EC_50_	Dilution	IC_50_	Dilution	EC_50_	Dilution	IC_50_	Dilution	EC_50_	Dilution	IC_50_	Dilution
Geometric mean (MG)	3.62	4,142.38	3.01	1,030.39	2.43	266.63	2.72	503.33	1.98	96.11	2.50	929.13
GCV (%)	**19.17**		**13.84**		**14.32**		**13.26**		**15.00**		**17.26**	

The numerical values highlighted in bold correspond to the final results considered for statistical analysis in each of the tests.

With respect to intermediate precision that represents the relative agreement when evaluating independent results within the same laboratory, assays with the JN.1 variant showed the greatest difference between GCVs. Additionally, the FRNT assay presented the highest GCV among all assays performed; however, the range of differences between assay increased compared to repeatability, ranging from 0.69% to 5.28%. All assays met the acceptance criterion of GCV < 30% (**Table 3**). We also observed that titers obtained by the FRNT assay were consistently higher than those obtained by the TCID_50_MN assay, regardless of the analyst. To confirm whether these differences were statistically significant and whether they were influenced by the analyst or the method, we performed a two-way ANOVA. This analysis demonstrated that for both the ancestral strain Wuhan strain across the three levels of serum (HLSP, *p* = 0.0004; MLSP, *p* ≤ 0.0001; LLSP, *p* ≤ 0.0001) and the XBB.1 variant (*p* ≤ 0.0001), both factors significantly influence the results; however, the interaction between them was not significant, indicating that their effects are independent. In contrast, the assay performed with the JN.1 variant did show significant differences between the analyst and the method (*p* = 0.0001).

**Table 3:** Table of intermediate precision results of the TCID_50_MN and FRNT assays for the ancestral strain and the Omicron subvariants with different serum pools.

Spike SARS-CoV-2 ancestral strain Wuhan												
Sample	HLSP				MLSP				LLSP			
Type of assay	TCID_50_MN		FRNT		TCID_50_MN		FRNT		TCID_50_MN		FRNT	
Units	EC_50_	Dilution	IC_50_	Dilution	EC_50_	Dilution	IC_50_	Dilution	EC_50_	Dilution	IC_50_	Dilution
Geometric mean (MG)	3.27	1,865.31	3.46	33,193.27	2.97	939.72	3.11	8,619.16	2.57	372.82	2.69	748.21
GCV (%)	**17.63**		**18.67**		**16.11**		**19.85**		**16.25**		**17.48**	

Spike SARS-CoV-2, omicron subvariants												
	BA.2		BA.5		XBB.1				JN.1			
Sample	V-LIT-POOL-3											
Type of assay	TCID_50_MN		TCID_50_MN		TCID_50_MN		FRNT		TCID_50_MN		FRNT	
Units	EC_50_	Dilution	IC_50_	Dilution	EC_50_	Dilution	IC_50_	Dilution	EC_50_	Dilution	IC_50_	Dilution
Geometric mean (MG)	3.65	4,473.27	3.10	1,257.48	2.48	301.95	2.81	632.30	1.97	92.84	2.64	3,992.66
GCV (%)	**18.89**		**15.21**		**14.48**		**13.79**		**16.88**		**22.16**	

The numerical values highlighted in bold correspond to the final results considered for statistical analysis in each of the tests.

### 3.3 Accuracy/bias

The Eurachem guide defines accuracy as an expression of the closeness of the mean of an infinite number of results from the evaluated method to a reference value. However, since it is not possible to perform an infinite number of measurements, accuracy *per se* cannot be determined; instead, it can be expressed quantitatively in terms of bias. Bias can be determined through recovery experiments using reference samples with an expected value (16, 19). The expected value of each of the evaluated pools was determined by previous TCID_50_MN and FRNT assays prior to validation with all spike protein variants. Validation results from both analysts (repeatability and intermediate precision) were compared with the expected value to determine bias and recovery percentage. Bias was expressed as the absolute difference between the expected value and the obtained average value obtained. Results showed higher bias values for analyst 1 compared to analyst 2 in the TCID_50_MN assay, whereas in the FRNT assay, bias distribution was more homogeneous between both analysts. Regarding recovery percentage, analyst 1 obtained higher values than analyst 2 in both the MN and FRNT assays, except for the JN.1 variant with the TCID_50_MN assays; these differences were not statistically significant. Therefore, in all cases, they met the previously established acceptance criteria (**Tables 4**, **5**).

**Table 4:** Trueness/bias results of the TCID_50_MN assay, for the ancestral strain and the Omicron subvariants with different serum pools.

Spike SARS-CoV-2 variant												
	Wuhan				BA.2		BA.5		XBB.1		JN.1	
Sample	HLSP		LLSP		V-LIT-POOL-3							
Reference value (EC_50_)	3.05		2.38		3.57		3.03		2.50		2.20	
Analyst	1	2	1	2	1	2	1	2	1	2	1	2
Average (EC_50_)	3.37	3.18	2.69	2.45	3.68	3.62	3.19	2.97	2.50	2.43	1.95	1.98
Standard deviation	0.20	0.27	0.16	0.18	0.27	0.28	0.18	0.14	0.15	0.16	0.27	0.18
Bias	0.31	0.11	0.31	0.07	0.11	0.05	0.16	0.02	0.04	0.07	0.25	0.22
Recovery (80% and 120%)	**110.05**	**103.55**	**111.74**	**102.80**	**103.21**	**101.32**	**105.15**	**99.44**	**101.43**	**97.12**	**88.82**	**90.18**

The numerical values highlighted in bold correspond to the final results considered for statistical analysis in each of the tests.

**Table 5:** Trueness/bias results of FRNT assay, for the ancestral strain and the Omicron subvariants with different serum pools.

Spike SARS-CoV-2 variant								
	Wuhan				XBB.1		JN.1	
Sample	HLSP		LLSP		V-LIT-POOL-3			
Reference value (IC_50_)	3.56		2.99		2.69		2.53	
Analyst	1	2	1	2	1	2	1	2
Average (IC_50_)	3.70	3.37	2.86	2.54	2.90	2.72	2.96	2.50
Standard deviation	0.53	0.14	0.18	0.18	0.09	0.12	0.68	0.24
Bias	0.14	0.19	0.13	0.45	0.21	0.03	0.43	0.03
Recovery (80% and 120%)	**103.91**	**94.58**	**95.81**	**84.92**	**107.81**	**101.01**	**116.89**	**98.97**

The numerical values highlighted in bold correspond to the final results considered for statistical analysis in each of the tests.

### 3.4 Robustness

The robustness of a method is the capacity to meet expected performance criteria during normal use; that is, the extent to which the method, and therefore the result, can be affected by small variations during its execution (16, 19). Therefore, we evaluated the effect of the adsorption time of the pseudovirus-sample mixture on the cell monolayer. Two conditions were tested: ±15 min relative to the standard assay conditions. The TCID_50_MN assay was performed with the ancestral strain, and the FRNT assay was performed with the ancestral strain as well as the XBB.1 and JN.1 Omicron subvariants. Twenty experimental replicates were performed with the corresponding sample, and the results were compared with those obtained in the repeatability test of each assay to calculate the recovery percentage. We observed that neither technique was affected by modifying adsorption time, with recovery percentages remaining within the range of 80% and 120% (**Table 6**).

**Table 6:** Robustness results of TCID_50_MN and FRNT assays for the ancestral strain Wuhan and Omicron subvariants XBB.1 and JN.1 with the different serum pools.

Spike SARS-CoV-2 variants								
	Wuhan*				XBB.1**		JN.1**	
Sample	HLSP		LLSP		V-LIT-POOL-3			
Reference value	3.56		2.99		2.69		2.53	
Analyst	1	2	1	2	1	2	1	2
Average	3.70	3.37	2.86	2.54	2.90	2.72	2.96	2.50
Standard deviation	0.53	0.14	0.18	0.18	0.09	0.12	0.68	0.24
Bias	0.14	0.19	0.13	0.45	0.21	0.03	0.43	0.03
Recovery (80% and 120%)	**103.91**	**94.58**	**95.81**	**84.92**	**107.81**	**101.01**	**116.89**	**98.97**

*Results obtained from the TCID_50_MN assay (EC_50_); **Results obtained from the FRNT assay (IC_50_).

The numerical values highlighted in bold correspond to the final results considered for statistical analysis in each of the tests.

### 3.5 Estimation of associated uncertainty

To ensure the reliability, comparability, and validity of results in a biological assay, uncertainty estimation is a key element in scientific and regulatory decision-making in internationally recognized accreditations (18, 22). **Supplementary Table 1** shows the complete procedure for estimating the associated uncertainty, considering all factors associated with the test result; from the mathematical model, the partial derivatives of each element were obtained, with which the combined uncertainty was calculated, multiplied by the coverage factor, to finally obtain the expanded uncertainty. Within the Spearman–Kärber mathematical model, X0 has an associated uncertainty, which involves elements associated with the method such as equipment, instruments, environment, analysts, and reagents, and is encompassed in the intermediate precision for the validation process of each variant. The SD per analyst and overall for each intermediate precision assay was considered as the typical measurement uncertainty. We managed to associate an expanded uncertainty value to each of the analysts and to the spike protein variant; with analyst 1, the uncertainty values were higher in all variants, except for the BA.5 variant (**Table 7**). Regarding the FRNT assay, the mathematical model used was the 4PL logistic regression model, considering the factors that affect the count of fluorescent focus and the calculation of the IC_50_ (**Supplementary Table 2**) with the Prims software version 10.3, as well as intermediate precision as a parameter that encompasses the factors associated with the measurand, as mentioned above with X0 in the TCID50MN test; also for this method, the standard deviation of each variable was also considered for the calculation, and since none remained constant, the values obtained by each analyst go through the same process, and the data for estimating the uncertainty were those obtained from the count done by the ImmunoSpot software, obtaining higher values with the ancestral Wuhan strain in all three levels of serum pools, compared to the Omicron subvariants XBB.1 and JN.1 (**Table 7**).

**Table 7:** Uncertainty results of TCID_50_MN and FRNT assays for the ancestral strain Wuhan and Omicron subvariants with different serum pools.

Associated uncertainty estimation: Spike SARS-CoV-2 Variants (TCID_50_MN)														
	Wuhan						BA.2		BA.5		XBB.1		JN.1	
Sample	HLSP		MLSP		LLSP		V-LIT-POOL-3							
TMU^1^ analyst	1	2	1	2	1	2	1	2	1	2	1	2	1	2
	0.34	0.25	0.24	0.21	0.24	0.19	0.37	0.29	0.17	0.20	0.24	0.15	0.54	0.29
TMU IP^2^	0.25		0.21		0.21		0.28		0.18		0.16		0.23	
CSU^3^	0.42	0.35	0.33	0.29	0.32	0.28	0.47	0.40	0.25	0.27	0.29	0.22	0.59	0.37
EU^4^	**0.84**	**0.71**	**0.66**	**0.59**	**0.64**	**0.57**	**0.93**	**0.81**	**0.50**	**0.54**	**0.58**	**0.44**	**1.17**	**0.74**

Associated uncertainty estimation: Spike SARS-CoV-2 Variants (FRNT)														
		Wuhan							XBB.1			JN.1		
Sample		HLSP		MLSP			LLSP		V-LIT-POOL-3					
TMU bottom^1^		22.63		10.24			24.53		10.61			19.26		
TMU top^2^		1.43		16.90			15.00		0.98			4.16		
TMU LogIC_50_^3^		0.38		0.48			0.32		0.08			0.23		
TMU Y^4^		24.63		24.79			20.34		24.89			26.56		
TMU HillSlop^5^		1.52		5.47			1.25		4.54			4.44		
TMU IP^6^		0.27		0.27			0.27		0.14			0.14		
CU^7^		0.48		0.56			0.45		0.16			0.28		
EU^8^		**0.96**		**1.11**			**0.91**		**0.33**			**0.55**		

^1^Typical measurement uncertainty of the minimum value of the response; ^2^TMU of the maximum value of the response; ^3^TMU of the maximum value of the negative logarithm of the IC_50_ value; ^4^TMU of the standard deviation of the IC_50_ value; ^5^TMU of the slope value; ^6^TMU of intermediate precision; ^7^Combined standard uncertainty; ^8^Expanded uncertainty.

**^1^**Typical measurement uncertainty; **^2^**TMU of intermediate precision; **^3^**Combined standard uncertainty; **^4^**Expanded uncertainty.

The numerical values highlighted in bold correspond to the final results considered for statistical analysis in each of the tests.

### 3.6 Assay quality internal control

As a complement to ensure the reliability of our results, we evaluated the stability of the TCID_50_MN assay and we performed random assays over a minimum period of 9 months, varying the cell line passage, analyst, pool aliquot (HLSP and V-LIT-Pool-3), and pseudoviral stock aliquot. We prepared a Levey–Jennings control chart, applying Westgard rules, and observed that no assay resulted in result rejection for exceeding three standard deviations during this time. This allowed us to detect and correct analytical deviations, validating the precision and accuracy of the measurements. Given the lack of international guidelines for this type of biological assay, these measurements can indirectly serve as a guide regarding the uncertainty obtained for biological assays of this nature, and establish an internal criterion generating an “objective uncertainty” based on the data obtained from this monitoring. Based on the results obtained and summarized in **Table 7**, for each of the analysts and variants of the spike protein, the expanded uncertainty (UE) data are within expected values given their nature and consistent with the readout systems used, such as the EC_50_ obtained from the cytopathic effect with the Spearman–Kärber formula in the TCID_50_MN assay and the IC_50_ calculated by the 4PL logistic regression mathematical model of the FRNT assay.

## 4 Discussion

Validated analytical methods are of essential importance in the research, development, and manufacture of products in the pharmaceutical and biopharmaceutical industry, because their main function is to provide reliable data with which decision-making is possible; however, these methods must be designed and suitable for use, as this approach reduces the risk of inconsistent results and imprecise decisions (25, 26). Since the beginning of the COVID-19 pandemic, there has been an accelerated development of therapeutic alternatives for detecting the causative agent and to evaluate the safety and protection of vaccines against the disease, using safe methods for VSV-based pseudovirus to measure nAbs such as those we report in this research, as well as those that have been reported since 2020 by other research groups, with their particularities such as the readout system (cytopathic effect, Luciferase, and GFP expression) (27–29). During the evaluation of new vaccines against any pathogen and/or disease, it is necessary to characterize the immune response of vaccinated individuals through blood samples, from which serum, plasma, and/or mononuclear cells can be obtained. This characterization should include the analysis of functional antibodies, description of the kinetics of the immune response, and factors that may affect it (15). The functional evaluation of antibodies aims to observe if, derived from immunization, the body is capable of producing antibodies that have some specific effect against the pathogen on *in vitro* studies; in the case of SARS-CoV-2, the evaluation of nAbs is of utmost importance because high titers are known to be associated with protection against symptomatic infection (30). As mentioned above, during evaluation of the COVID-19 vaccines, several methodologies with different readout systems have been reported for the quantification of nAbs, which yield differential values with respect to the protection thresholds, that is why, in order to homogenize these results, an international standard serum was established by the WHO (31, 32). Different research groups have described validation processes for the quantification of nAbs; in this context, we are convinced that it is not enough to describe the method in detail; a series of regulatory requirements must also be met, not only to demonstrate the consistency of the method, but mainly to guarantee the reliability of the results considering the factors associated with estimating uncertainty; the objective is to generate tools with a significant differentiating value at the clinical level for the establishment of the correlates of protection, as well as for decision-making for the approval of vaccine proposals by international organizations (33–35). We standardized and validated two neutralization assays using the same VSV-based pseudovirus platform that expresses variants of the SARS-CoV-2 spike protein, with two different readout systems TCID50MN and FRNT, to evaluate nAbs similar to those used during the evaluation of COVID-19 vaccines and the establishment of standard serums by the WHO.

During the validation process, we found that both assays are equally reliable to correctly differentiate between positive and negative samples, indicating that analytical performance is within the confidence intervals of statistical test as reported previously by others (36). We also demonstrated that both assays are robust; that is, they are not affected by variations in the time of virus–sample interaction. On the other hand, although differences were observed in terms of the variability of the assay, as well as in the bias that the assay may generate between analysts, there was no statistically significant impact on the result obtained, meeting the established acceptance criteria. Overall, the TCID_50_MN technique showed less variability than that of FRNT, although it showed lower sensibility than in FRNT, indicating that the latter has greater sensitivity due to the readout system, as reported by Puglia and collaborators and others (33, 37). However, in this research, we report GCV values lower than those reported by other groups, relative to the established limit of 30% for both biological assays (35, 37, 38). Another important point to consider regarding the difference in titers obtained with different assays is the dilution factor. Although it may not seem to be a factor, subtle differences in titers can be masked or overestimated. For example, Huang et al. reported greater consistency of results with a 1:3 dilution factor than with 1:5 (38). While previous reports have addressed the validation process of neutralization assays with high analytical performance, none considered uncertainty estimation. We are the first research group to report the uncertainty estimates for both assays, contributing to strengthening the validation parameters of a biological assay, guaranteeing the reliability and accuracy of the results in terms of regulatory requirements with international validity.

## 5 Conclusions

It is true that one of the limitations of these methodological proposals is that we use a pseudovirus system and not viral isolates; however, the system based on the VSV vector proved to be consistent in the results obtained, being a cost-effective biotechnological alternative. The validation of TCID_50_MN and FRNT microneutralization assays for detecting and quantifying nAbs against SARS-CoV-2 variants marks a significant advancement in the diagnostic and regulatory capabilities of laboratories. Both methods demonstrated high sensitivity, specificity, and robustness, meeting international standards of quality and technical competence as outlined in ISO/IEC 17025:2017. The integration of pseudovirus systems and the estimation of associated uncertainty strengthen the reproducibility and reliability of results, which are key elements for evaluating correlates of protection and conducting population-level surveillance.

Although the FRNT assay yielded higher titers and acceptable inter-analyst variability, both methods are complementary and offer versatile platforms for immunological studies. Sustained implementation of quality controls and analytical standardization further reinforces the utility of these assays in clinical and regulatory contexts. This work establishes a validated framework for quantifying nAbs, contributing to vaccine development and the precise characterization of immune responses to emerging SARS-CoV-2 variants.

## Data Availability

The original contributions presented in the study are included in the article/**Supplementary Material**. Further inquiries can be directed to the corresponding author.

## Supplementary material

Supplementary Table 1 The entire procedure for estimating uncertainty of the TCID_50_MN neutralization assay.

Supplementary Table 2 The entire procedure for estimating uncertainty FRNT neutralization assay.
