# Supplementary material for: Associated uncertainty estimation during the validation process of TCID50 and FRNT neutralization assays against SARS-CoV-2 variants, used in population surveillance research and correlates of protection
Source: Front Immunol. 2026 Mar 18;17:1768395. doi: 10.3389/fimmu.2026.1768395 (PMC13038988; doi:10.3389/fimmu.2026.1768395)
Supplement: Supplementary Table 1 — The entire procedure for estimating uncertainty of the TCID50MN neutralization assay. [file DataSheet1.pdf]

Table S1. Uncertainty Components and Calculation of Expanded Uncertainty for the TCID50MN Assay

1. Acceptance Criteria

**Cellular Control:** Intact cell monolayer with typical epithelial morphology	**Viral Control:** Cell monolayer showing cytopathic effect	**Positive Sample:** $EC_{50} > 1.78$, dilution 1:60	**Negative Sample:** $EC_{50} < 1.78$, dilution 1:60

2. Mathematical Model

$$log_{10} = \left(x_0 + \frac{d}{2} + d\sum \frac{r_i}{n_i}\right)$$

3. Identification of Sources of Uncertainty

Sources of Uncertainty		Associated Sources of Uncertainty				
Log_{10} of the Reciprocal of the Minimum Dilution at which All Wells Exhibit Protection	LX0	Micropipette	Reading of the Plates at the End of the Assay	Microscope	Incubator	Virus Titer
Intermediate Precision	PI					
Analyst	Analyst 1 and 2					

4. Quantification of Components of the Standard Measurement Uncertainty $u(x_i)$

Description	Code	Type of Uncertainty Evaluation	Standard Measurement Uncertainty	Remarks
Log_{10} of the reciprocal of the minimum dilution at which all wells show protection	X_0	A	**0.2500**	
Log_{10} of the dilution factor (3)	d		**0.0000**	The value is constant, as the method was validated with a dilution factor of 3
Number of wells used in each dilution (2)	n_i		**0.0000**	The value is constant, as the method was validated with a constant number of wells per dilution = 2
Number of wells showing protection (among n_i)	r_i		**0.0000**	
Intermediate Precision	PI		**0.2500**	

5. Combined Standard Measurement Uncertainty uc(yi)

Formulas	Sensitivity Coefficient (C_i)			Standard Measurement Uncertainty	$uc(y_i)$
Sensitivity Coefficient $c_i = \frac{\partial y}{\partial x}$	$\frac{\partial y}{\partial x_0}$	-1	**-1.0000**	**-0.2500**	**0.3536**
	$\frac{\partial y}{\partial r}$	$-\sum_{i=0}^{K}\frac{1}{n_i}$	**-0.5000**	**0.0000**	
Combined Standard Uncertainty $u_c(y_i) = \sqrt{\sum_{i=1}^{N}(\frac{\partial y}{\partial x} * u(x_i)^2}$	$\frac{dy}{dd}$	$\frac{1}{2}-\sum_{i=0}^{K}\frac{r_i}{n_i}$	**-0.5000**	**0.0000**	
	$\frac{dy}{dn}$	$\sum_{i=0}^{K}\frac{r_i}{(n_i)^2}$	**-0.5000**	**0.0000**	

6. Expanded Uncertainty (U)

**Coverage Factor k***	**2**

Formula

$$U = u_c(y_i)\ x\ k$$

7. U

0.7071

WorkFlow for estimating the Expanded Uncertainty of the analytical method. Step 1, Definition of the measurand (Accpetance criteria); Step 2, Model the process (Matemathical model); Step 3, Identification of Sources of Uncertainty; 4, Quantification of Components of the Standard Measurement Uncertainty $u(x_i)$; 5, Calculate the Combined Standard Measurement Uncertainty uc(yi); 6, Calculate the Expanded Uncertainty (U); 7, Express the result in the units of the method.
