# Supplementary material for: Associated uncertainty estimation during the validation process of TCID50 and FRNT neutralization assays against SARS-CoV-2 variants, used in population surveillance research and correlates of protection
Source: Front Immunol. 2026 Mar 18;17:1768395. doi: 10.3389/fimmu.2026.1768395 (PMC13038988; doi:10.3389/fimmu.2026.1768395)
Supplement: Supplementary Table 2 — The entire procedure for estimating uncertainty FRNT neutralization assay. [file DataSheet2.pdf]

Table S2. Uncertainty Components and Calculation of Expanded Uncertainty for the FRNT Microneutralization Assay with FFU

1. Acceptance Criteria

**Cellular Control:** Intact cell monolayer with typical epithelial morphology and average FFU<5	**Viral Control:** Cell monolayer showing cytopathic effect and FFU count between 16 and 314	**Positive Sample:** EC_{50} > 1.78, dilution 1:60	**Negative Sample:** EC_{50} < 1.78, dilution 1:60

2. Mathematical Model

$$x = logIC50 - \left[\frac{log\left(\frac{S-I}{Y-I}-1\right)}{M}\right]$$

3. Identification of Sources of Uncertainty

Sources of Uncertainty		Associated Sources of Uncertainty				
Lower (Minimum or Baseline Value of the Response)	I	Analyst	Micropipette	ImmunoSpot	Incubator	Reading of the Plates at the End of the Assay
Upper (Maximum Value or Response Plateau)	S					
Negative Logarithm of IC50	LogIC50					
Slope	M					
Neutralization Percentage	Y					
Intermediate Precision	PI					

4. Quantification of Components of the Standard Measurement Uncertainty $u(x_i)$

Description	Code	Type of Uncertainty Evaluation	Standard Measurement Uncertainty	Remarks
Lower (Minimum or Baseline Value of the Response)	I	A	**22.6258**	The value corresponds to the standard deviation of the minimum or baseline response values
Upper (Maximum Value or Response Plateau)	S		**1.4279**	The value corresponds to the standard deviation of the maximum response values
Negative Logarithm of IC50	LogIC50		**0.3801**	The value corresponds to the standard deviation of the negative logarithm of the IC50 value
Neutralization Percentage	y		**24.6278**	The value corresponds to the standard deviation of the inhibition percentage above and below the IC50
Slope	M		**1.5171**	The value corresponds to the standard deviation of the slope obtained from the curve
Intermediate Precision	PI		**0.2712**	The value corresponds to the standard deviation of the PI from the verification

5. Combined Standard Measurement Uncertainty uc(yi)

Formulas	Sensitivity Coefficient (C_i)			Standard Measurement Uncertainty	$uc(y_i)$
$c_i = \frac{\partial y}{\partial x}$ ficient	$\frac{\partial x}{\partial I}$	$\frac{\partial x}{\partial I} = -\frac{1}{M * \ln(10) * (y - I)}$	**-0.0051**	**-0.1151**	**0.4810**
	$\frac{\partial x}{\partial I}$	$\frac{\partial x}{\partial S} = \frac{1}{M * \ln(10) * (S - Y)}$	**0.0052**	**0.0074**	
$u_c(y_i) = \sqrt{\sum_{i=1}^{N}(\frac{\partial y}{\partial x} * u(x_i))^2}$ nty	$\frac{\partial x}{\partial LogIC50}$	$\frac{\partial x}{\partial logIC50} = 1$	**1.0000**	**0.3801**	
	$\frac{\partial x}{\partial y}$	$\frac{\partial x}{\partial y} = \frac{S - I}{M * \ln(10) * (S - Y) * (Y - I)^2}$	**0.0003**	**0.0070**	
	$\frac{\partial x}{\partial M}$	$\frac{\partial x}{\partial M} = \frac{log\left(\frac{S-I}{Y-I} - 1\right)}{M^2}$	**-0.0014**	**-0.0022**	

7. Expanded Uncertainty (U)

**Coverage Factor k***	**2**

Formula

$$U = u_c(y_i)\ x\ k$$

7. U

0.9620

WorkFlow for estimating the Expanded Uncertainty of the analytical method. Step 1, Definition of the measurand (Accpetance criteria); Step 2, Model the process (Matemathical model); Step 3, Identification of Sources of Uncertainty; 4, Quantification of Components of the Standard Measurement Uncertainty $u(x_i)$; 5, Calculate the Combined Standard Measurement Uncertainty uc(yi); 6, Calculate the Expanded Uncertainty (U); 7, Express the result in the units of the method.
